# Enhancing the antigenicity and immunogenicity of monomeric forms of hepatitis C virus E2 for use as a preventive vaccine

**DOI:** 10.1074/jbc.RA120.013015

**Published:** 2020-04-16

**Authors:** Rob J. Center, Irene Boo, Lilian Phu, Joey McGregor, Pantelis Poumbourios, Heidi E. Drummer

**Affiliations:** ‡ Burnet Institute, 85 Commercial Road, Melbourne 3004, Australia; § Department of Microbiology and Immunology at the Peter Doherty Institute for Infection and Immunity, University of Melbourne, Melbourne 3000, Australia; ¶ Department of Microbiology, Monash University, Clayton 3056, Australia

**Keywords:** hepatitis C virus (HCV), vaccine development, glycoprotein, disulfide, immunogenicity, antigen engineering, broadly neutralizing antibody (bNAb), D123-HMW, multimerization, protein refolding

## Abstract

The E2 glycoprotein of hepatitis C virus (HCV) is the major target of broadly neutralizing antibodies (bNAbs) that are critical for the efficacy of a prophylactic HCV vaccine. We previously showed that a cell culture–derived, disulfide-linked high-molecular-weight (HMW) form of the E2 receptor–binding domain lacking three variable regions, Δ123-HMW, elicits broad neutralizing activity against the seven major genotypes of HCV. A limitation to the use of this antigen is that it is produced only at low yields and does not have a homogeneous composition. Here, we employed a sequential reduction and oxidation strategy to efficiently refold two high-yielding monomeric E2 species, D123 and a disulfide-minimized version (D123A7), into disulfide-linked HMW-like species (Δ123r and Δ123A7r). These proteins exhibited normal reactivity to bNAbs with continuous epitopes on the neutralizing face of E2, but reduced reactivity to conformation-dependent bNAbs and nonneutralizing antibodies (non-NAbs) compared with the corresponding monomeric species. Δ123r and Δ123A7r recapitulated the immunogenic properties of cell culture–derived D123-HMW in guinea pigs. The refolded antigens elicited antibodies that neutralized homologous and heterologous HCV genotypes, blocked the interaction between E2 and its cellular receptor CD81, and targeted the AS412, AS434, and AR3 domains. Of note, antibodies directed to epitopes overlapping with those of non-NAbs were absent. The approach to E2 antigen engineering outlined here provides an avenue for the development of preventive HCV vaccine candidates that induce bNAbs at higher yield and lower cost.

## Introduction

Hepatitis C virus (HCV)^2^ is a significant global health problem. Approximately 71 million people are chronically infected with the virus, which causes progressive liver disease, including cirrhosis and cancer, that can ultimately be fatal or treatable only by liver transplant. Treatment with direct acting antivirals mediates high levels of viral clearance but does not prevent reinfection, and the fact that many infected individuals are unaware of their HCV-positive status leads to ongoing viral transmission. Modeling suggests that timely HCV elimination would be facilitated by the combined actions of direct acting antivirals and a yet to be developed preventive vaccine (1, 2).

HCV is an enveloped, positive-sense, single-stranded RNA virus. The viral surface glycoprotein E2 mediates attachment to target cell receptors, including the major receptor CD81, and is the main target for neutralizing antibodies (NAbs). Crystallographic data show that soluble E2 has a globular structure with a central immunoglobulin β-sandwich flanked by front and back layers (3–5). E2 has two broad antigenic regions: (i) a neutralizing face comprised of the front layer and CD81 binding loop targeted by NAbs and (ii) a nonneutralizing face comprised of sections of the back layer and immunoglobulin β-sandwich targeted by nonneutralizing antibodies (non-NAbs).

Spontaneous viral clearance, which occurs in ∼30% of infected individuals, has been correlated with the early development of NAbs that have broad reactivity against multiple HCV isolates (bNAbs) and broadly reactive cell-mediated immunity (CMI) (6, 7). Furthermore, passively infused monoclonal bNAbs or polyclonal antibodies derived from HCV-infected humans can provide protection from challenge in small-animal models of HCV infection (8–12). A number of vaccine development approaches based on the elicitation of bNAbs and/or CMI, including recombinant protein, virus-like particles, and vaccine vectors, have been assessed in animal models or phase I and II clinical trials. The responses elicited have in most cases shown limited cross-genotype reactivity (reviewed in Refs. 7 and 13), and no HCV vaccine candidate aimed at developing bNAbs has advanced beyond a phase I clinical trial.

The development of a broadly protective HCV vaccine has been challenging for a number of reasons. Hepatitis C has extremely high sequence variability due to the lack of proofreading function of the virally encoded RNA-dependent RNA polymerase. As a result, HCV circulates as eight divergent genotypes with median within- and between-genotype amino acid sequence divergences of 23 and 33%, respectively (14). A prophylactic vaccine must therefore provide broad protection against the global pool of circulating viruses. Other viral defense mechanisms that blunt the immune response to E2 include the high number of attached glycans, some of which surround the CD81-binding site and have been demonstrated to shield bNAb epitopes (15, 16). E2 contains three variable domains. Hypervariable region 1 (HVR1) is a target of type-specific NAbs but plays a nonessential role in viral entry and so rapidly develops escape mutations that further diversify the viral sequence pool (17–20). A further role for HVR1 is to maintain E2 in a conformation that is resistant to neutralization (21–23). Hypervariable region 2 (HVR2) and the intergenotypic variable region also play roles in reducing accessibility of the CD81-binding site and NAb epitopes (24). Together, these factors present a challenge to vaccine development.

**Figure 1.**
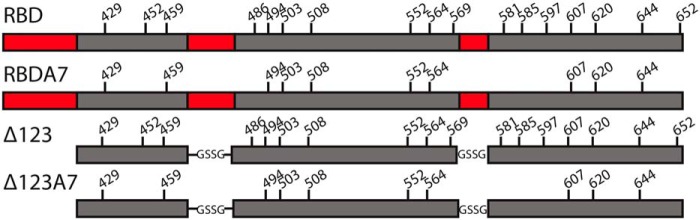
**Schematic representation of E2 antigens.** Hypervariable regions 1 and 2 and the intergenic variable region are shown in *red*. In Δ123 and Δ123A7, N-terminal truncation removed hypervariable region 1, whereas hypervariable region 2 and the intergenic variable region were replaced with short linkers (amino acids GSSG). The positions of Cys residues are indicated with residue numbers *above* the schematic. In RBDA7 and Δ123A7, Cys residues at positions 452, 486, 569, 581, 585, 597, and 652 were mutated to alanine.

We previously reported on a recombinant, soluble version of the E2 glycoprotein from which HVR1, HVR2, and the intergenotypic variable region were removed from the receptor-binding domain (RBD) (Δ123, Fig. 1). Like its WT RBD counterpart, the proteins expressed in mammalian cell culture include monomeric species as well as heterogeneous disulfide-linked forms. Enhanced cross-genotype neutralizing responses were preferentially generated in guinea pigs vaccinated with a high-molecular-weight form (Δ123-HMW) (25). Distinguishing Δ123-HMW from monomeric Δ123 was an occluded nonneutralizing surface and the preferential generation of antibodies that overlap with AS412, AS434, and AR3. A limitation to the use of cell culture–derived Δ123-HMW is that it comprises less than 5% of the total Δ123 yield and contains impurities, both significant problems in terms of cost and ease of purification for scaled-up vaccine production.

In this study, we used sequential reduction and oxidation to drive disulfide-bond rearrangement in order to refold monomeric E2 into an HMW-like form. We applied such refolding to RBD, Δ123, and their variants in which 7 cysteine residues were mutated to alanine (Δ123A7 and RBDA7; Fig. 1), which leads to a potentially simplified intramolecular disulfide-bonding pattern and a relatively homogeneous monomeric profile (26). We succeeded in refolding up to 70% of Δ123 and Δ123A7 monomers into assembled HMW-like forms (Δ123r and Δ123A7r) and compared the biophysical and antigenic properties of the assembled and cell culture–derived HMW forms. In addition, the immunogenicity was assessed in guinea pigs. Δ123r and Δ123A7r largely recapitulated the immunogenic properties of cell culture–derived Δ123-HMW and present a new avenue for the production of vaccine candidates with enhanced immunogenicity for HCV.

## Results

### Soluble E2 monomers can be refolded into higher-molecular-weight forms

**Figure 2.**
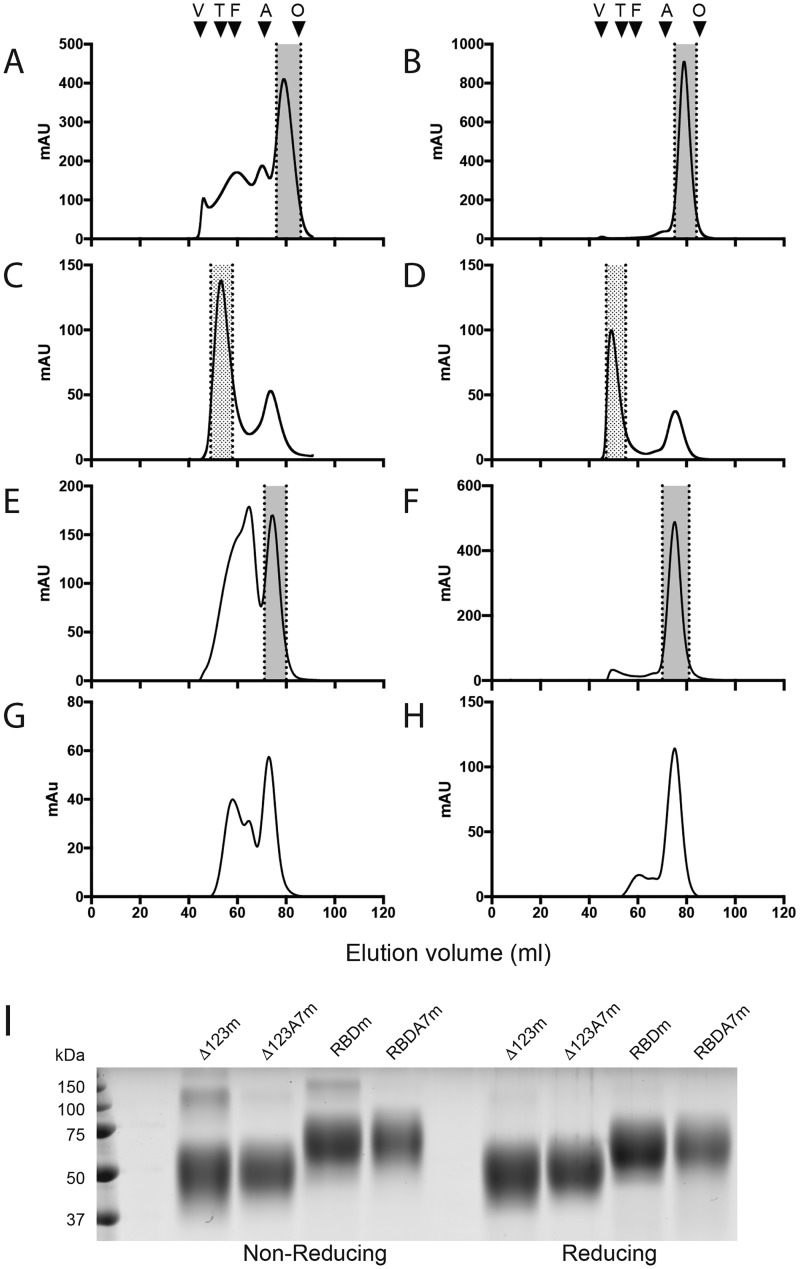
**SEC profiles of Δ123 (*A*), Δ123A7 (*B*), assembled Δ123 (Δ123r) (*C*), assembled Δ123A7 (Δ123A7r) (*D*), RBD (*E*), RBDA7 (*F*), assembled RBD (*G*), and assembled RBDA7 (*H*).** SEC was performed using a 16/600 Superdex 200 column. The *shaded area* between the *dotted lines* in *A*, *B*, *E*, and *F* represents the monomeric fractions that were pooled and used in subsequent analyses and immunization studies or were subjected to refolding by DTT treatment. The *stippled area* in *C* and *D* represents the assembled fractions that were pooled and used in subsequent analyses and immunization studies. The SEC standards (abbreviation, elution volume, and mass) used were blue dextran (*V* (void), 45 ml, >2,000 kDa), thyroglobulin (*T*, 52 ml, 669 kDa), ferritin (*F*, 59 ml, 440 kDa), aldolase (*A*, 70 ml, 158 kDa), and ovalbumin (*O*, 84 ml, 43 kDa). *I*, SDS-PAGE of the indicated E2 monomers in the presence or absence of reducing agent.

The formation of Δ123-HMW during expression in 293-F cells is driven by the formation of intermolecular disulfide bonds. However, this multimeric form generally represented less than 5% of the total purified glycoprotein and contains impurities (Table 1). We sought to improve the efficiency of production and homogeneity of HMW through limited reduction of intramolecular disulfide bonds in E2 monomers followed by slow oxidation to promote the assembly of higher-order species through the formation of intermolecular bonds, while preserving the immunogenicity of the molecule and its potential utility as a vaccine candidate. Affinity-purified Δ123 and Δ123A7 showed strikingly different size-exclusion chromatography (SEC) profiles. Δ123 consisted of a range of species with peaks at 46-, 60-, 70-, and 79-ml volume (4, 31.5, 16.5, and 48% of the total, respectively) (Fig. 2*A*), corresponding to the previously described HMW1, HMW2, dimer, and monomer species (25). In contrast, Δ123A7 was almost entirely monomeric (Fig. 2*B*). SEC fractions corresponding to monomeric Δ123 and Δ123A7 (indicated by the *gray shading* in Fig. 2, *A* and *B*) were pooled and concentrated, and monomeric status was confirmed by analytical SEC (Fig. S1, *A* and *B*). In both reducing and nonreducing SDS-PAGE, the monomeric forms of Δ123 and Δ123A7 migrated to positions consistent with their expected monomer glycoprotein size of ∼47 kDa (25), confirming the lack of stable intermolecular disulfide bonds (Fig. 2*I*). Yields of ∼20–40 and 10–15 mg of purified monomeric protein per liter of tissue culture supernatant were obtained for cells stably transfected with Δ123 and transiently transfected with Δ123A7, respectively. SEC of the monomers after DTT-induced refolding showed that both Δ123 and Δ123A7 efficiently assembled HMW species with ∼60–70% of the total in this form (Fig. 2 (*C* and *D*, respectively) and Table 1). The HMW peak for Δ123A7 eluted slightly earlier than that of Δ123 (49 ml compared with 53 ml). Fractions corresponding to the refolded species (indicated by the *hatched shading* in Fig. 2, *C* and *D*) were pooled and used for further analyses. Similar to Δ123 and Δ123A7, RBD eluted as a range of species with a distinct monomeric peak at 75 ml, whereas RBDA7 was almost entirely monomeric (Fig. 2, *E* and *F*). The SEC fractions corresponding to monomeric RBD and RBDA7 (Fig. 2, *E* and *F*, *gray shading*) were pooled and concentrated, and monomeric status was confirmed by analytical SEC (Fig. S1, *C* and *D*). These species ran to positions consistent with their expected monomer glycoprotein size of ∼55 kDa in SDS-PAGE (Fig. 2*I*). When monomeric RBD and RBDA7 were subject to DTT-induced refolding, assembled HMW was formed less efficiently, with a lower percentage of the total refolding and smaller size of the HMW species generated (Fig. 2 (*G* and *H*, respectively) and Table 1). This indicated that the presence of one or more of the HVRs inhibited DTT-induced refolding; hence, RBD and RBDA7 were not analyzed further.

**Table 1:** **Typical proportion (percentage of total) of the different species purified from 293-F tissue culture supernatant after separation of multimeric species by SEC, as determined by area under the curve analysis using version 7 Unicorn software**

	Cell culture–derived HMW	Monomer	Assembled HMW	Residual monomer after assembly
Δ123	<5	47–51	60–72	28–40
Δ123A7	<1	95–100	56–70	29–44
RBD	<1–8	22–34	40	50
RBDA7	<5	91–97	10	90

### Biophysical characterization of refolded Δ123 and Δ123A7

Biophysical techniques were used to examine the size of refolded Δ123r and Δ123A7r. We previously used SEC-multiangle light scattering (SEC-MALS) analysis to show that monomeric Δ123 was 47 kDa and cell culture–derived Δ123-HMW was ∼2,400 kDa, whereas a smaller species, HMW2, was 240 kDa (25). SEC-MALS analysis of assembled Δ123r and Δ123A7r proteins revealed that they were polydispersed with a wide molar mass range, with both having a weight average molar mass of 409 kDa (Table 2). This was ∼9-fold higher than monomeric Δ123, but smaller than that previously reported for cell culture–derived Δ123-HMW (25).

**Table 2:** **Tabulated SEC-MALS analysis of Δ123r and Δ123A7r** SEC fractions corresponding to HMW peaks after refolding were pooled and concentrated prior to SEC-MALS analysis.

Sample	Retention time	Molar mass range	Weight average molar mass
	*min*	*kDa*	*kDa*
Δ123r	12.5–15.5	774–210	409
Δ123A7r	12–14.5	675–262	409

**Figure 3.**
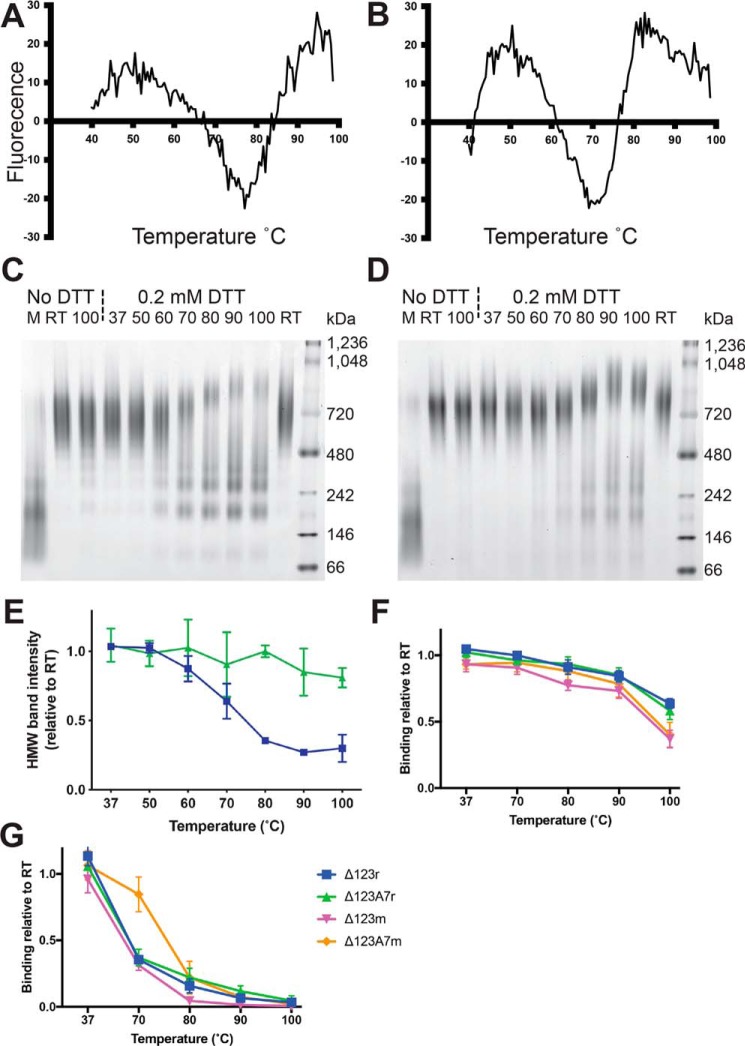
**Assessment of thermal stability of monomeric and assembled E2 antigens.** DSF of monomeric Δ123 (*A*) and monomeric Δ123A7 (*B*). BN-PAGE of Δ123r (*C*) and Δ123A7r (*D*) under nondenaturing conditions either with no heating (RT) or with pretreatment at the indicated temperature for 5 min in the presence or absence of the 0.2 mm DTT. *M*, monomer prior to refolding. Signal intensity of HMW bands analyzed by BN-PAGE in the presence of 0.2 mm DTT at the indicated temperatures relative to RT + 0.2 mm DTT, which was assigned a value of 1.0 (*E*). ELISA reactivity of MAb14 (*F*) and HC84.27 (*G*) after pretreatment of E2 antigens at the indicated temperatures for 30 min prior to coating. Titers were obtained by interpolating fitted curves at 25-fold above background (given by reactivity to BSA) and were expressed relative to that of RT, which was assigned a value of 1.0. *Error bars* in *E* represent the S.D. of two independent experiments, and *error bars* in *F* and *G* represent the S.D. of three independent experiments. Note that in one case each for Δ123 monomers and Δ123A7 monomers, the threshold for titration to HC84.27 after treatment at 100 °C was not met, in which case they were assigned a titer of the highest concentration of antibody used (3,160 ng/ml).

We next examined the thermal stability of the E2 antigens using differential scanning fluorimetry (DSF). The traces obtained for monomeric Δ123 and Δ123A7 (Fig. 3, *A* and *B*) indicated moderate differences in thermal stability with melting temperature (*T_m_*) values of 77 and 71 °C, respectively. This suggested that the lower number of cysteine residues and consequently reduced number of disulfide bonds in Δ123A7 reduced thermal stability compared with Δ123. We were unsuccessful in obtaining *T_m_* values for the assembled glycoproteins using DSF, probably due to excess uptake of dye prior to heating. We therefore utilized indirect methods to assess the thermal stability of these molecules. A modification of blue native PAGE (BN-PAGE) was used to assess the resistance to dissociation of the multimeric structure of the assembled antigens by heating to temperatures ranging from room temperature (RT) to 100 °C for 5 min either in the absence or presence of the reducing agent DTT prior to BN-PAGE. In the absence of pretreatment, both Δ123r and Δ123A7r migrated to ∼720 kDa. The multimeric structure of the antigens was largely (Δ123r, Fig. 3*C*) or completely (Δ123A7r, Fig. 3*D*) resistant to heating to 100 °C in the absence of reducing agent, consistent with the cross-linking of subunits with nonlabile disulfide bonds. The addition of 0.2 mm DTT during heating to 60 °C and above caused progressive dissociation of Δ123r HMW-like multimers, with a mean HMW band intensity at ∼720 kDa of 0.88 at 60 °C reducing to 0.27 and 0.30 at 90 and 100 °C, respectively, relative to DTT treatment at RT (Fig. 3, *C* and *E*). In contrast, Δ123A7r HMW-like multimers were more resistant to dissociation at the same temperatures and DTT concentration, with mean HMW band intensities at ∼720 kDa of 0.85 and 0.81 at 90 and 100°C, respectively, relative to RT (Fig. 3, *D* and *E*).

Thermal stability of specific epitopes was analyzed using the conformation-dependent nonneutralizing MAb14 (24) in a direct ELISA modified by the additional step of heating the antigens at the indicated temperature in carbonate buffer for 30 min prior to coating the plates. MAb14 was used as it binds equally to Δ123, Δ123A7, and the refolded versions of these antigens (Table 3). Results are shown as MAb14 binding to treated antigen, relative to untreated antigen. The MAb14 epitope was largely resistant to thermal disruption up to 90 °C, with 100 °C treatment reducing the binding of monomeric proteins marginally more than the refolded versions (Fig. 3*F*). The bNAb HC84.27 was also used in this assay as it is well-characterized and binds the neutralizing face of E2, has a discontinuous epitope (27), and showed adequate binding to all of the antigens assessed (Table 3). HC84.27 binding was more sensitive to thermal disruption than MAb14, with binding being markedly reduced by treatment at temperatures of 80 °C or above for all antigens assessed (Fig. 3*G*). The HC84.27 epitope was more resistant to heat treatment up to 70 °C within the Δ123A7 monomer compared with other antigens.

**Table 3:**
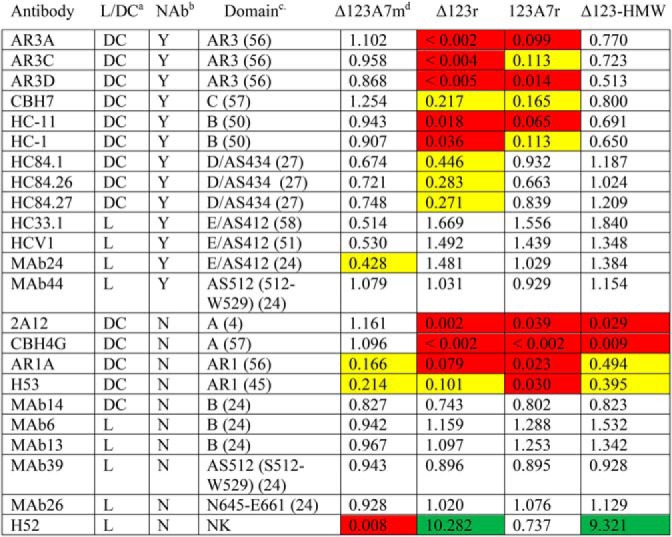
**Antigenicity of monomeric and assembled Δ123 and Δ123A7 and cell culture-derived Δ123-HMW measured by direct ELISA** Numbers show the fraction of antibody reactivity compared with that of monomeric Δ123, which was assigned a reactivity of 1.0 for all antibodies. Indicated are relative binding of < 0.5 (yellow shading), < 0.1 (red shading), and > 2.0 (green shading).

*^a^* L, linear; DC, discontinuous.

*^b^* Neutralizing activity.

*^c^* Epitope domain targeted (reference). NK, not known. For consistency, the Ser512-Trp529 domain is referred to as AS512.

*^d^* Δ123A7 monomer.

### Antigenic comparison of refolded and monomeric E2

Monomeric and assembled forms of Δ123 and Δ123A7 and cell culture–derived Δ123-HMW were compared for their reactivity with a panel of E2-specific mAbs by direct ELISA (Fig. S2), with the -fold difference in binding compared with Δ123 monomer shown in Table 3. Compared with the other antigens, Δ123r and Δ123A7r showed markedly reduced reactivity to bNAb HC11 (domain B), and AR3 bNAbs AR3A and AR3D, with Δ123r also showing markedly reduced reactivity to AR3C and HC-1 (domain B). These data suggest that a subset of conformation-dependent epitopes are occluded or their structure is altered on the neutralizing face of E2 in the assembled glycoproteins. By contrast, the reactivity of Δ123r and Δ123A7r was similar to cell culture–derived Δ123-HMW and monomeric antigen forms for bNAbs with linear epitopes localizing to the neutralizing face of E2, including epitope I/domain E/AS412 (HC33.1, HCV1, and MAb24), domain D/AS434 (HC84.27), and the CD81-binding loop (MAb44), and to nonneutralizing antibodies MAb6, MAb13, MAb26, and MAb39. Both Δ123r and Δ123A7r showed markedly reduced binding to the non-NAbs 2A12, CBH4G, and AR1A compared with the corresponding monomers. Cell culture–derived Δ123-HMW also had markedly reduced binding to 2A12 and CBH4G, suggesting occlusion of the nonneutralizing face of E2 in the cell culture–derived HMW and assembled glycoproteins. The H52 mAb was an exception in that binding to Δ123r was strongly enhanced, recapitulating the enhanced binding of this mAb to the cell culture–derived Δ123-HMW. This antibody is sensitive to mutation at Cys^652^,^3^ and as a consequence, H52 binding to monomeric Δ123A7 and Δ123A7r was either markedly or moderately reduced, respectively, compared with monomeric Δ123.

We sought to confirm the direct ELISA binding data by using biolayer interferometry (BLI) to measure the reactivity of the analyte-phase E2 antigens to a subset of mAbs (HCV1 (AS412), AR3C (AR3), and 2A12 (domain A)). In these experiments, the multimeric forms of antigen did not have measurable off rates in most cases, presumably through avidity effects, precluding obtaining *K_D_* values (Fig. S3 and Table S1). The *K_D_* values obtained for Δ123 and Δ123A7 monomers for the three antibodies were broadly similar, supporting the direct ELISA data that showed similar binding levels of these antigen/antibody combinations. BLI sensorgrams of Δ123r/AR3C and Δ123A7r/AR3C and all multimeric antigens to 2A12 showed minimal binding, consistent with the minimal binding seen by direct ELISA.

**Figure 4.**
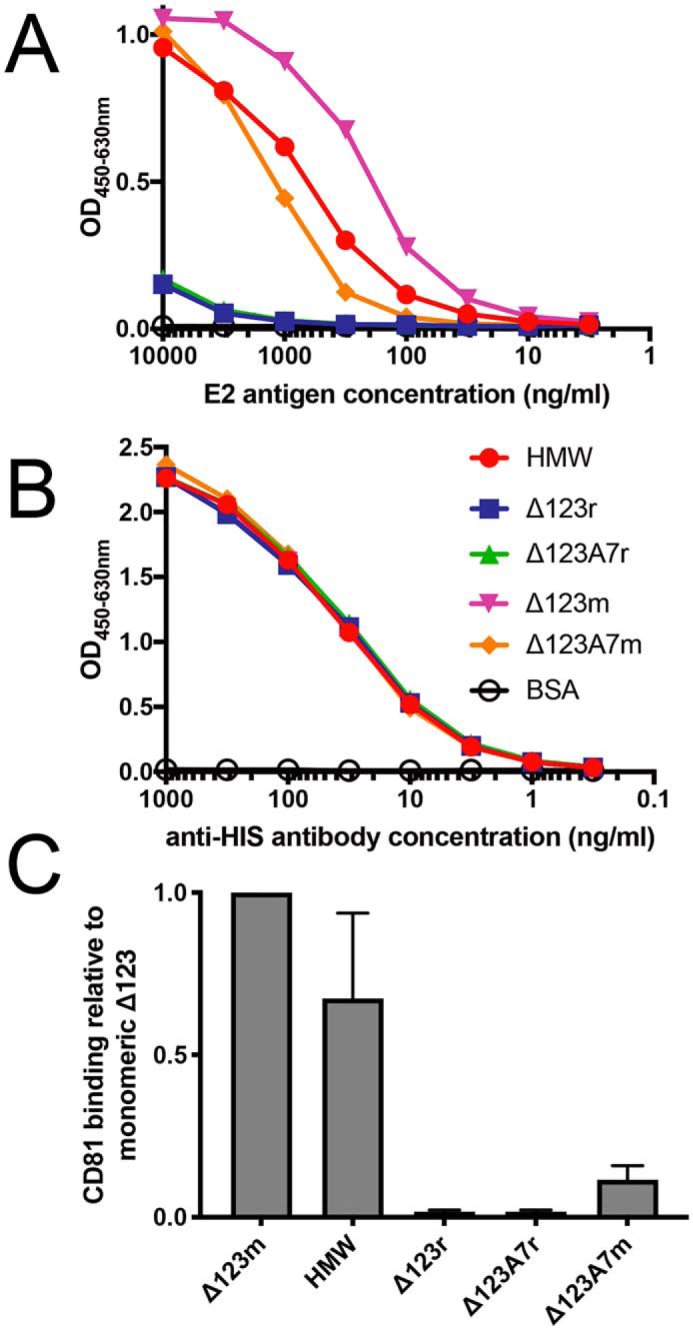
**Comparison of the binding of E2 antigens to CD81.** The ability of plate-bound CD81 LEL to interact with the indicated concentrations of E2 antigen was measured by capture ELISA, using an anti-His_6_ tag antibody to detect E2 (*A*). Equal reactivity to the anti-His_6_ tag antibody confirmed equal loading of all E2 antigens in the direct ELISA (*B*). CD81 LEL binding of E2 antigens relative to Δ123m is shown. Titers were obtained by interpolation using a value of 20-fold above background (defined by the binding of BSA), with Δ123 monomer binding being assigned a value of 1.0 (*C*). *Error bars* in *C* represent the S.D. of three independent experiments.

We next examined the ability of the different E2 species to bind to the plate-bound large extracellular loop (LEL) of CD81 in a capture ELISA. As reported previously, cell culture–derived Δ123-HMW showed an ∼2–3-fold reduction in LEL binding compared with Δ123 monomers (25). Assembled Δ123r and Δ123A7r did not bind to CD81 LEL at levels significantly above background (Fig. 4, *A* and *C*). The binding of CD81 LEL to Δ123A7 monomers was reduced by approximately 1 log compared with Δ123 monomers, suggesting that the mutational loss of 7 cysteine residues reduces CD81 binding capacity. Binding of the anti-His_6_ mAb was used to confirm equal loading of E2 antigens in a direct ELISA (Fig. 4*B*). CD81 binding was also assessed by coating plates with E2 antigen and measuring the capture of CD81 LEL (data not shown). This experiment showed similar relative binding of different antigen species to CD81 LEL, including the loss of reactivity of Δ123r and Δ123A7r.

### Assembled forms of E2 induce strong E2-specific antibody responses

**Figure 5.**
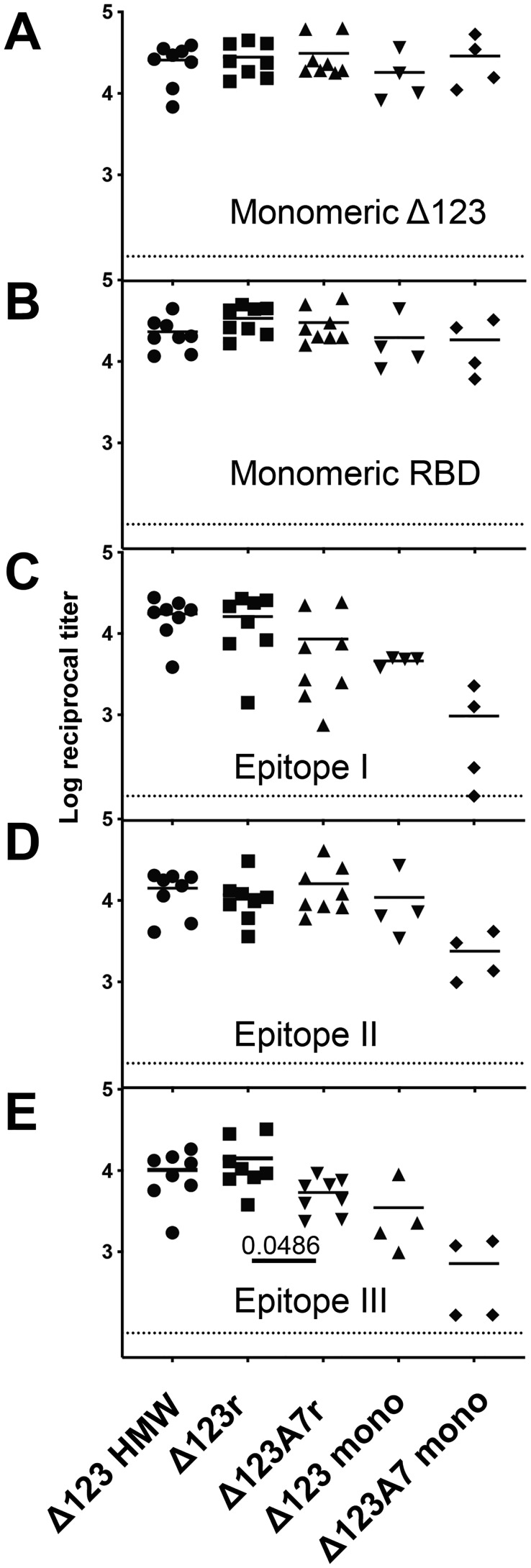
**ELISA binding titers of guinea pig serum antibodies to monomeric Δ123 (*A*), monomeric RBD (*B*), and peptides corresponding to epitopes I (*C*), II (*D*), and III (*E*) of the H77c strain.** Half-log serial dilutions of sera were performed, and curves were fitted by nonlinear regression. Titers were obtained by interpolation using a value of 25-fold above background (defined by signal in the absence of sera) for the Δ123 and RBD antigens and 20-fold above background (defined as above) for peptides I, II, and III. The *dashed line* shows the lower detection limit of the assay (1:100 dilution).

To assess the immunogenicity of the assembled Δ123r and Δ123A7r proteins, guinea pigs were immunized four times with the proteins in the MF59-analog adjuvant AddaVax^TM^. The E2-specific titers of the sera of guinea pigs vaccinated with Δ123-HMW (*n* = 8, group 1), Δ123r (*n* = 8, group 2), Δ123A7r (*n* = 8, group 3), Δ123 monomers (*n* = 4, group 4), Δ123A7 monomers (*n* = 4, group 5), and negative controls (*n* = 3, group 6) toward the monomeric forms of Δ123 or RBD were determined by direct ELISA (Fig. 5, *A* and *B*, respectively). Antibody titers were robust and similar for all immune groups toward both antigens, generally ranging from 10^4^ to 10^5^. Within the Δ123-HMW, Δ123r, and Δ123A7r groups, where animal numbers were sufficient to support statistical analysis, there were no significant differences between the groups (*p* > 0.05), and within-group means had a narrow range between 10^4.2^ and 10^4.6^.

To examine whether antibodies able to recognize epitopes I, II, and III were generated, the corresponding avidin-bound biotinylated peptides were used to capture specific antibodies present in the immune sera (Fig. 5, *C–E*, respectively). All animals generated measurable antibodies specific to these regions with the single exception of one serum from the Δ123A7 monomer group against epitope I. There were generally similar titers of antibodies elicited in the Δ123-HMW, Δ123r, and Δ123A7r immune groups with a trend toward lower titers in animals that received monomeric immunogens, particularly Δ123A7 monomers. Within the Δ123-HMW, Δ123r, and Δ123A7r groups, the only significant difference was that the mean titer against epitope III for the Δ123A7r-vaccinated group was narrowly significantly lower (*p* = 0.0486) than the Δ123r-vaccinated group.

### Vaccine-induced antibodies compete with CD81 LEL and mAbs for binding to E2

**Figure 6.**
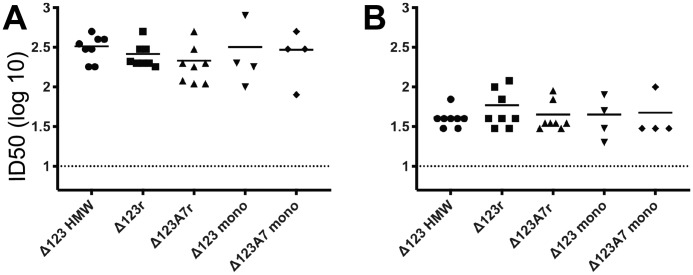
**The ability of guinea pig sera to compete with homologous H77c RBD and heterologous G2a JFH-1 RBD for binding to CD81 LEL.** Half-log serial dilutions of sera and a constant concentration (0.5 μg/ml) of H77c RBD (*A*) and JFH-1 RBD (*B*) were mixed, incubated for 1 h, and then added to plate-bound CD81 LEL in a competitive ELISA. E2 antigen was detected using the anti-His_6_ tag antibody. Curves were fitted by nonlinear regression, and ID_50_ values were interpolated using binding in the absence of guinea pig sera as 100% binding. Data are shown as the log_10_ ID_50_ of individual guinea pig sera. The *dashed line* shows the lower detection limit of the assay (1:10 dilution).

To examine whether the E2-vaccinated groups generated antibodies able to prevent the interaction between the homologous genotype 1a RBD and CD81, an ELISA was performed in which RBD and immune sera were mixed in solution and incubated prior to addition to plate-bound CD81 LEL. The immune sera from all E2-vaccinated animals competed with the interaction between CD81 and the homologous G1a RBD antigen, with similar titers elicited between groups (Fig. 6*A*), despite significant occlusion of the CD81 surface in the case of Δ123r and Δ123A7r. Antibodies able to block the interaction between heterologous genotype 2a RBD and CD81 LEL were also present in all sera, albeit at lower titers (Fig. 6*B*). There were no statistically significant differences between the groups assessed for either interaction.

**Figure 7.**
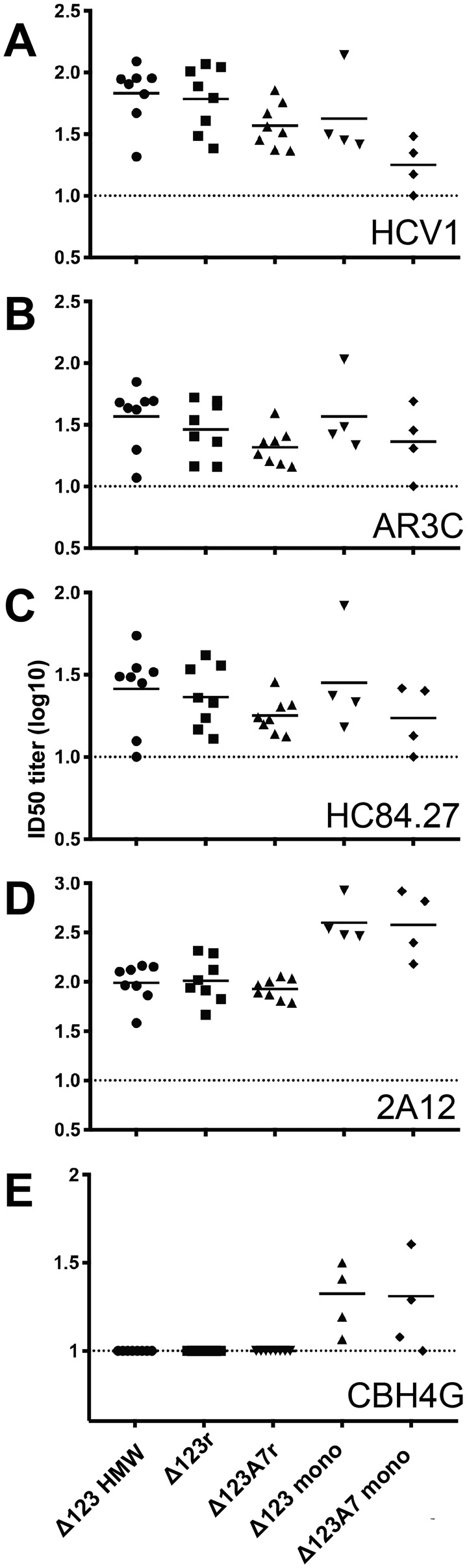
**The ability of guinea pig sera to compete with E2- specific mAb for binding to monomeric RBD.** Half-log serial dilutions of sera and constant concentrations of HCV1 (100 ng/ml) (*A*), AR3C (50 ng/ml) (*B*), HC84.27 (250 ng/ml) (*C*), 2A12 (250 ng/ml) (*D*), and CBH4G (100 ng/ml) (*E*) were incubated with plate-bound RBD in a competitive ELISA. mAb binding was detected using a horseradish peroxidase–conjugated secondary antibody specific for human antibody. Curves were fitted by nonlinear regression, and ID_50_ values were interpolated using binding in the absence of guinea pig sera as 100% binding. Data are shown as the log_10_ ID_50_ of guinea pig sera. The *dashed line* shows the lower detection limit of the assay (1:10 dilution).

We also examined the specificity of the immune serum by employing a competitive ELISA using a subset of the bNAbs and non-NAbs that were used to assess antigenicity of the E2 molecules. Immune sera of all vaccinated animals were able to compete with bNAbs HCV1 (AS412), AR3C (AR3), and HC84.27 (AS434) for interaction with the homologous RBD. Where group sizes allowed statistical comparison (Δ123-HMW, Δ123r, and Δ123A7r), there were no statistically significant differences between the groups. There was a trend toward higher titers in the Δ123-HMW, Δ123r, and Δ123 monomer groups compared with the Δ123A7r and Δ123A7 monomer groups (Fig. 7, *A–C*). In contrast, sera from animals vaccinated with monomeric Δ123 and Δ123A7 had higher titers of antibodies able to compete with binding of the non-NAb 2A12 and CBH4G compared with the sera from animals vaccinated with the cell culture–derived or assembled HMW forms (Fig. 7, *D* and *E*). In fact, no CBH4G competing antibodies were observed for any of the animals in the groups that were vaccinated with Δ123-HMW, Δ123r, or Δ123A7r. Overall, these results show that the assembled immunogens elicit antibodies that overlap with bNAb epitopes located in antigenic regions AS412, AS434, and AR3, even when the antigenic reactivity to these mAbs was markedly reduced in the case of AS434 and AR3 and the immunogenicity of non-NAb epitopes was significantly decreased.

### Assembled forms of E2 induce neutralizing antibodies

**Figure 8.**
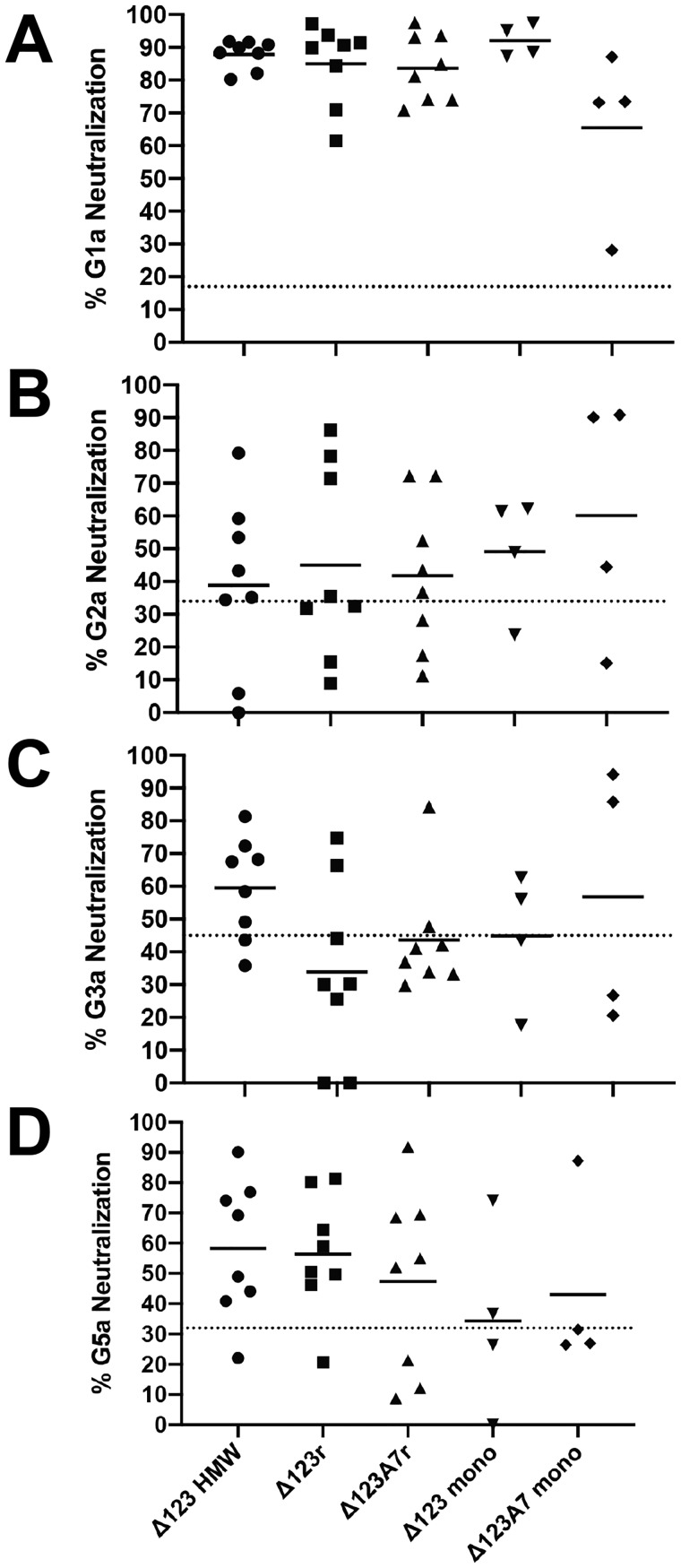
**Neutralization of HCV by guinea pig sera.** The percentage neutralization mediated by a 1:40 dilution of each individual animal serum against HCV G1a (H77pp) (*A*), G2a (J6cc) (*B*), G3a (S52cc) (*C*), and G5a (SA13cc) (*D*) is shown. Individual *data points* are the mean of within-assay triplicate measurements, and *bars* represent within-group means. Where negative neutralization values were obtained, they were assigned a value of 0. The *dotted line* represents the mean level of nonspecific neutralization of three control sera from guinea pigs vaccinated with adjuvant alone.

Neutralization assays were performed on 1:40 dilutions of all sera against both homologous genotype 1a using pseudotyped retroviral particles (HCVpp) and heterologous cell culture–derived virus (HCVcc) containing the structural regions of genotypes 2a, 3a, and 5a (Fig. 8). Where group sizes allowed statistical comparison (Δ123-HMW, Δ123r, and Δ123A7r), no statistically significant differences were found; however, we noted several trends in these data. The strongest levels of neutralization were detected toward homologous G1a HCVpp by all immune groups, with only one serum from the Δ123A7 monomer group failing to achieve 50% neutralization. Heterologous neutralization was strongest toward genotype 5a with 4 of 8, 5 of 8, and 5 of 8 sera reaching 50% neutralization in the Δ123-HMW, Δ123r, and Δ123A7r groups respectively. For genotype 2a, 3 of 8, 3 of 8, and 3 of 8, and for genotype 3a, 5 of 8, 2 of 8, and 1 of 8 reached 50% neutralization in the Δ123-HMW, Δ123r, and Δ123A7r groups, respectively. In the combined monomer groups, 4 of 8, 4 of 8, and 2 of 8 neutralized genotype 2a, 3a, and 5a at the 50% level, respectively.

We next sought to determine whether neutralizing activity correlated with other ELISA binding or inhibitory titer parameters combining the immune sera across all vaccination groups (Table 4 and Fig. S4). Most parameters had a statistically significant positive correlation to H77 neutralization, the exceptions being epitope III–binding titer, inhibition of the G2a RBD/CD81 interaction, and inhibition of the interaction between RBD and the non-NAbs 2A12 and CBH4G. The strongest positive correlations with neutralization were observed for the inhibition of the binding of the bNAbs HCV1 (AS412), AR3C (AR3), and HC84.27 (AS434) and CD81 LEL to H77c RBD and for the direct binding titers to epitopes I (AS412) and II (AS434) (*p* < 0.005, *r* > 0.5 for these parameters). This suggests that HMW and assembled HMW forms of E2 are able to elicit antibodies targeting multiple neutralization domains, including AS412, AS434, and AR3, and reduce the generation of potentially deleterious non-Nabs.

**Table 4:** **Correlation of H77pp neutralization with other experimental parameters using the Spearman *r* test** A boldface *p* value denotes a significant correlation.

Experimental parameter	*r*	*p*
RBD monomer binding titer	0.3662	**0.0393**
Δ123 monomer binding titer	0.4549	**0.0089**
Epitope I binding titer	0.5308	**0.0018**
Epitope II binding titer	0.5176	**0.0024**
Epitope III binding titer	0.2232	0.2194
CD81/G1a inhibition	0.512	**0.0027**
CD81/G2a inhibition	0.03119	0.8654
HCV1/G1a inhibition	0.6243	**0.0001**
AR3C/G1a inhibition	0.5337	**0.0017**
HC84.27/G1a inhibition	0.5125	**0.0027**
2A12/G1a inhibition	0.09971	0.5872
CBH4G/G1a inhibition	−0.07549	0.6813

## Discussion

Here, we report on efforts to synthetically produce a disulfide-linked HMW multimer of the HCV E2 glycoprotein using sequential reduction and oxidation to drive intermolecular disulfide bond formation. This was prompted by our previous finding that an HMW-Δ123 multimer, which was spontaneously formed during expression in 293-F cells, showed superior immunogenicity compared with monomeric E2 but was expressed at very low levels. Stably transfected 293-F cell clones yielded 20–40 mg of Δ123 monomer/liter of tissue culture supernatant, of which ∼60–70% could be assembled into the HMW form by sequential reduction and oxidation. This compares with the less than 5% of the total yield for cell culture–derived Δ123-HMW. The refolding strategy was efficient at producing HMW multimers of Δ123 and Δ123A7 but not RBD or RBDA7, suggesting that the presence of the HVRs sterically interferes with intermolecular disulfide bond formation and/or maintenance of assembled multimers. To efficiently form HMW complexes, Δ123 and Δ123A7 monomers would have undergone extensive intramolecular disulfide bond breakage and then formation of intermolecular disulfide bonds when sequentially reduced and oxidized during the assembly process. The disruption of intramolecular disulfide bonds within the monomeric antigens and/or the formation of novel intramolecular disulfide bonds that were not present prior to refolding would be expected to broaden the range of conformational states adopted by Δ123r and Δ123A7r. This may account for the reduced reactivity of the assembled forms to a number of conformation-dependent bNAbs and non-NAbs and soluble CD81 that was observed. In contrast, reactivities to linear mAbs were similar when assembled and monomeric forms were compared. Despite this apparent global skewing toward the presentation of linear epitopes, Δ123r and Δ123A7r elicited antibodies that competed with the interaction between RBD and CD81 and the conformational bNAbs tested that targeted the AR3 and AS434 epitopes to a similar extent as sera generated by monomeric E2. Importantly, antibodies raised against assembled Δ123r and Δ123A7r and cell culture–derived Δ123-HMW either did not compete with the two non-NAbs assessed or did so less potently than antibodies present in sera raised against monomeric Δ123 and Δ123A7. The occlusion of non-Nab epitopes may refocus the immune system toward NAb targets. This concept is well-established in HIV vaccine development, where mutations have been designed to stabilize the HIV Env trimer and occlude non-NAb epitopes with some success in eliciting NAb responses in small animals (28, 29).

It has been shown that functional E2 on the surface of viral particles exists as a noncovalently linked heterodimer with E1 (30) (and references therein). One study also found that virus-associated E1 forms homotrimers (31), suggesting a form comprised of a trimer of homodimers. The potential higher-order quaternary structure of these proteins on viral particles is less well-characterized. One study found that virion-associated E1 and E2 formed disulfide-linked HMW complexes of greater than 440 kDa with evidence to suggest that this form was functional (32). It is possible that the HMW form of E2 in our study retains a subset of conformations present in virus-associated E2. More efficient antigen uptake and presentation of larger molecules by antigen-presenting cells and/or more avid binding to B-cell receptors may also have played a role in the enhanced immunogenicity of HMW E2. This is well-recognized for large-sized antigen platforms, such as virus-like particles or liposomes, or the coupling of small antigens to larger carrier molecules but has been less well-studied for single-component multimeric complexes. Larger multimeric forms of the lipopolysaccharide O antigen of *Francisella tularensis* and the meningococcal capsular polysaccharide of *Neisseria meningitidis* have been shown to mediate enhanced immunogenicity compared with smaller forms of the same antigen (33, 34).

A number of viral glycoproteins have a high degree of structural plasticity in some protein domains, and this is believed to play a role in viral immune evasion. It has been shown that stabilizing HIV-1 envelope protein structure by glutaraldehyde-mediated cross-linking selectively enhances the humoral immune responses to key neutralizing epitopes (35). Structural plasticity has also been reported for HCV E2 (36, 37) despite E2 being highly stable overall, as evidenced by a high melting temperature. This is exemplified by the 412–423 N-terminal region, which displays high sequence conservation, contributes to the CD81-interactive region, and is a target epitope of bNAbs. Crystal-derived structures of a number of bNAb-derived Fabs in complex with peptides corresponding to this region of E2 showed that the peptide variously adopted either a β-hairpin (21, 38–41), extended (42), or short antiparallel β-sheet/extended coil (43) conformation. DSF analysis showed that Δ123 and to a lesser extent Δ123A7 monomers had high overall thermostability, which has previously been reported for similar E2 constructs using calorimetry (37). The post-heating ELISA binding data indicated that the MAb14 epitope was generally resistant to disruption. Assembled versions of E2 were slightly more heat-resistant than the corresponding monomers, which may be an advantage in terms of immune recognition of normally labile epitopes. In comparison, the HC84.27 epitope was more heat-sensitive, with the Δ123A7 monomers showing more heat resistance than Δ123 monomers or either assembled HMW forms. It therefore appears that thermal resistance can be epitope-dependent. It is likely that reformation of individual epitopes occurs after heat treatment in the ELISA experiments, whereas this would not occur during DSF, where protein unfolding is measured continuously in real time. If Δ123A7 was able to refold more efficiently through a pathway that was simplified by the reduced number of disulfide bonds, this may explain why it had a lower *T_m_* in DSF but showed higher heat stability by ELISA at the HC84.27 epitope. In BN-PAGE experiments, multimers of Δ123A7r showed greater resistance to dissociation than Δ123r when samples were heated in the presence of DTT prior to electrophoresis, in contrast to the DSF data showing that monomeric Δ123A7 was less heat-stable than Δ123 monomers. A simplified assembly may have allowed Δ123A7r to form more compact multimers that were more resistant to disassociation by treatment with heating and the reducing agent DTT than Δ123r.

When used to immunize guinea pigs, the HMW forms (cell culture–derived HMW, Δ123r, and Δ123A7r) elicited similar RBD- and Δ123-binding titers as the monomeric forms of these glycoproteins. When titers against three peptides corresponding to CD81 interactive regions/NAb targets (epitopes I–III) were compared, there was a trend for Δ123 monomers and especially Δ123A7 monomers to generate lower titers than the HMW forms. These data are consistent with the maintenance of reactivity of HMW to antibodies with linear epitopes seen in the antigenicity studies (Table 3 and Fig. S2) and suggest a possible bias toward the induction of antibodies with linear epitopes. They are also consistent with the reduced antibody/HMW binding off rates observed with BLI, which may translate to more avid B cell receptor binding and prolonged signaling. The individual immune sera generated by all antigens consistently and robustly neutralized the homologous G1a H77c pseudotyped viral particles. Heterologous G5a was more frequently neutralized by sera generated by the HMW forms, with 14 of 24 (58%) sera showing at least 50% neutralization compared with 2 of 8 (25%) sera of monomer-vaccinated animals. G2a and G3a were generally less consistently neutralized, with 50% or less of the sera neutralized to the 50% level in most immunization groups. G1a neutralization was also consistently positively correlated to most binding parameters, including competition with bNAbs but not non-NAbs for binding to RBD monomers. Not unexpectedly, given the less consistent neutralization seen for G2a, G3a, and G5a, few significant positive correlations between the neutralization of these viruses and binding parameters were observed (data not shown).

Overall, we found that assembled versions of Δ123 when used in combination with AddaVax^TM^ were as effective as cell culture–derived HMW at generating antibodies that bind intact E2 or peptides corresponding to neutralizing targets, block the interaction between E2 and CD81 or bNAbs (but not non-NAbs), and neutralize virus. The HMW assembly strategy described here was very efficient at producing disulfide-linked HMW at levels compatible with vaccine production compared with the very low yields of cell culture–derived HMW. This novel approach shows utility for production of HCV vaccine candidates and may have broader vaccine applicability where the advantages associated with larger antigen size are sought.

## Experimental procedures

### Recombinant protein expression and purification

The soluble HCV E2 ectodomain comprising amino acids 384–661 (RBD) (H77c polyprotein numbering used here and throughout), the Δ123 E2 core domain in which the three HVRs were either removed (residues 384–408) or replaced with GSSG linkers (residues 461–485 and 570–580) and modified versions of these glycoproteins bearing seven cysteine-to-alanine mutations (A7: C452A, C486A, C569A, C581A, C585A, C597A, and C652A) (Fig. 1) were expressed in Freestyle 293-F cells (293-F, Thermo Fisher Scientific) as described previously (25, 26). Δ123 was produced using a stable transfected cell clone, whereas Δ123A7, RBD, and RBDA7 were produced in cells transiently transfected using 293fectin (Thermo Fisher Scientific) according to the manufacturer's recommendations. All versions were purified from tissue culture supernatant by affinity chromatography using Talon resin (Clontech, Mountain View, CA) via the C-terminal His_6_ tag following the manufacturer's guidelines. Eluates were concentrated and buffer-exchanged to PBS adjusted to pH 6.8 (PBS 6.8) and subjected to SEC using a Superdex 200 16/600 column (GE Healthcare, Uppsala, Sweden). Analytical SEC to confirm the isolation of monomeric E2 was performed using a Superdex 200 10/300 column (GE Healthcare). CD81 LEL was expressed and purified as a dimer in *Escherichia coli* as described previously (44).

### Antibodies

The human monoclonal antibodies AR1A, AR3A, and AR3D (45) were gifts from Mansun Law (Scripps Institute), and CBH7 (46), HC-11 (47), HC84.26 (48), CBH4G (46), and HC-1 (49, 50) were gifts from Steven Foung (Stanford University). The mAbs AR3C (45), HC84.1 (27, 48), HC84.27 (27, 48), 2A12 (4), and HCV1 (51) were produced in-house by co-transfecting 293-F cells with corresponding human immunoglobulin heavy and light chain–expressing vectors and recovering antibody from conditioned tissue culture media using Protein G-Sepharose beads. In the case of 2A12, the murine variable regions of this mAb were fused with the human immunoglobulin chains to form a humanized antibody. Murine mAbs H52 and H53 (52) were gifts from Jean Dubuisson (Pasteur Institute) and Harry Greenberg (Stanford University), and WEH3.3H3.188 (anti-His_6_ tag mAb) was a gift from Catherine Owczarek (CSL, Melbourne). The murine mAb series MAb6, MAb13, MAb14, and MAb24 were raised against Δ123, and MAb26, MAb39, and MAb44 were raised against RBD. These mAbs were used as concentrated hybridoma supernatants with additional purification in the case of MAb24 and MAb44 using Protein G–Sepharose beads as described previously (24).

### Assembly of HMW-like E2 proteins

E2 monomers were buffer-exchanged from PBS 6.8 to 50 mm carbonate-bicarbonate buffer, pH 9.6, at a final E2 concentration of 1 mg/ml. DTT was added to a final concentration of 0.6 mm, followed by incubation at 37 °C for 30 min. The DTT concentration was then adjusted to 1.2 mm followed by further incubation at 37 °C for 30 min. PBS 6.8 equaling 50% of the reaction volume was added followed by incubation at RT for 15 min to allow for slow disulfide bond reformation. This step was repeated twice with the amount of PBS 6.8 added equaling 50% of the original reaction volume on each occasion. The reaction buffer was then fully exchanged back into PBS 6.8 and concentrated prior to SEC.

### PAGE

A modification of the BN-PAGE method was performed in the presence of the indicated concentration of the reducing agent DTT and/or with sample heating at the indicated temperature prior to electrophoresis. Native PAGE 4–16% BisTris gels (Invitrogen) were used following the manufacturer's instructions. The indicated E2 antigens (4 μg) and NativeMark protein standards (Thermo Fisher Scientific) were adjusted to 1× sample buffer (4× sample buffer: 200 mm BisTris, 64.2 mm HCl, 200 mm NaCl, 40% (w/v) glycerol, 0.004% (w/v) Ponceau S) prior to loading on the gel. The gel was fixed in 50% ethanol and 2% phosphoric acid; stained in 8.5% phosphoric acid, 10% ammonium sulfate, 20% methanol, and 0.12% Coomassie blue G-250 dye; and imaged using a LI-COR Odyssey IR imager and version 3.0 software. Band intensity was quantified with Image Lab version 6 software (Bio-Rad). Denaturing SDS-PAGE of the indicated E2 monomeric antigens (4 μg) and Precision Plus protein standards (Bio-Rad) was performed using standard conditions either in the absence or presence of the reducing agent, β-mercaptoethanol. Gels (12% Tris/glycine) were stained with Coomassie dye and imaged as above.

### DSF

The thermal stability of E2 antigens was tested by diluting 10 μg of protein into a 25-μl volume with 5× SYPRO Orange Protein Gel Stain (Thermo Fisher Scientific) in duplicate. The samples were then heated in an Mx300 qPCR System (Agilent Technologies) using the Stratagene MX PRO program in 0.5 °C increments, starting at 25 °C and ending at 95 °C for 1 min/temperature step. Fluorescence was read at the end of each increment in triplicate. Excitation was at 492 nm, and emission was at 610 nm. The *T_m_* in °C was determined to be the minimum of the negative first derivative of the melting curve.

### SEC-MALS

SEC-MALS was performed as described previously (25).

### Immunizations

Guinea pigs (outbred tricolor) that were matched for gender, weight, and age were immunized subcutaneously with 100 μg of E2 protein in PBS 6.8 in a 1:1 (v/v) mix with AddaVax^TM^ adjuvant (InvivoGen, San Diego, CA) four times at 3-week intervals. A negative control group was immunized as above with a 1:1 (v/v) mix of PBS 6.8 and adjuvant. Two weeks after the final dose, blood was collected by terminal cardiac puncture and allowed to clot for serum preparation. Sera were stored at 4 °C, with heat inactivation at 56 °C for 30 min prior to use in the case of the neutralization assays. Animals were housed and all procedures were performed at the Preclinical, Imaging, and Research Laboratories, South Australian Health and Medical Research Institute (Gilles Plains, Australia). All animal experiments were performed in accordance with the eighth edition of the Australian Code for the Care and Use of Animals for Scientific Purposes and were approved by the SAHMRI Animal Ethics Committee, project number SAM210.

### ELISA

#### 

##### Direct ELISA

The relative reactivity of E2 antigens to mAbs was assessed by ELISA as described previously (24) except that E2 (250 ng/well) was directly coated onto the plastic surface. Half-log serial dilutions of mAbs were incubated for 1 h and detected using horseradish peroxidase–labeled antibody (Dako, Glostrup, Denmark) against the appropriate primary antibody species. Color reactions were measured with a Multiskan Ascent plate reader (Thermo Electron, Waltham, MA). mAb binding to different antigens was compared by fitting curves with nonlinear regression using Prism version 7 software, and titers were obtained by interpolation of optical density (OD) values 20-fold above that of background, as defined by binding to BSA. Binding was then expressed as -fold difference compared with monomeric Δ123. The relative reactivity of guinea pig serum antibodies to the indicated E2 antigens was also determined by direct ELISA as described above. A cut-off OD value of 25-fold above background, as defined by signal in the absence of sera, was used to determine the dilution titer for each individual guinea pig serum.

##### Capture ELISA

To determine the relative reactivity of E2 antigens to CD81, ELISA plates were coated with CD81 LEL, blocked, and incubated with serial dilutions of E2 antigens for 2 h. The amount of E2 antigen captured was measured using an anti-His_6_ mAb. Reactivity of guinea pig sera to peptides based on H77c sequences for epitope I (^408^KQNIQLINTNGSWHINSTALN^428^), epitope II (^430^NESLNTGWLAGLFYQHKFNSSG^451^) and epitope III (H77c, ^523^GAPTYSWGANDTDVFVLNNTRPPLGNW^549^) were also determined by capture ELISA. Plate-bound avidin was used to capture the biotinylated peptide (1 μg/ml for 1 h) followed by the addition of serial dilutions of guinea pig sera and subsequent steps as outlined under “Direct ELISA.” In this case, a cut-off OD value of 20-fold above background (defined by signal in the absence of sera) was used to determine the titer.

##### Competitive ELISA

The ability of antibodies within immune sera to compete with mAbs or CD81 LEL for binding to monomeric RBD was measured in antibody competition or E2-CD81 inhibition assays as described previously (25). Inhibitory titers were expressed as the reciprocal dilution of immune serum that reduces the binding reaction being competed by 50% (inhibitory dilution 50, ID_50_) using binding in the absence of sera as 100% binding.

### BLI

BLI-based measurements were determined using an Octet RED System (ForteBio, Fremont CA). Antibodies were diluted in 1× kinetic buffer to 10 μg/ml and immobilized onto anti-human IgG Fc capture biosensors (ForteBio). Kinetics assays were carried out at 30 °C using standard kinetics acquisition rate settings (5.0 Hz, averaging by 20) at a sample plate shake speed of 1,000 rpm. The kinetic experiments included five steps: (*a*) baseline (180 s); (*b*) antibody loading (300 s); (*c*) second baseline (180 s); (*d*) association of antigen (600 s), and (*e*) dissociation of antigen (900 s). Fitting curves were constructed using ForteBio Data Analysis 10.0 software using a 1:1 binding model, and double reference subtraction was used for correction.

### Neutralization assays

HCV neutralization assays were performed as described previously (53). Briefly, HEK293T cells were co-transfected in a 1:1 (w/w) ratio of pE1E2H77c and pNL4–3.LUC.R-E- to produce HCV H77pp (54, 55). 1:40 dilutions of guinea pig sera were added to H77pp and incubated for 1 h at 37 °C before addition to Huh7.5 cells. After incubation for 4 h, the inocula were removed, and cells were incubated in fresh media for 72 h. Following lysis in cell culture lysis buffer (Promega, Madison WI), luciferase activity in clarified lysates was measured by using a luciferase substrate (Promega) on a CLARIOstar microplate reader fitted with luminescence optics (BMG Lab Technologies). Infectious cell culture–derived genotype 2a (J6), 3a (S52), and 5a (SA13) HCVcc were produced by transfecting Huh7.5s with *in vitro*–transcribed RNA by electroporation as described previously (25). NAb assays were performed by mixing HCVcc with 1:40 dilutions of guinea pig sera as described above with incubation for 42 h after removal of the inocula. Luciferase activity in cell lysates was measured using *Renilla* luciferase substrate (Promega).

### Statistics

Statistical between-group comparisons of guinea pig sera were performed where group size was sufficient (*n* = 8, Δ123-HMW, Δ123r, and Δ123A7r groups). Curves were fitted by nonlinear regression using one-site–specific binding with Hill slope. Data were statistically compared using the nonparametric Kruskal-Wallis test with Dunn's multiple comparisons. Correlations between parameters were tested using the nonparametric Spearman test and combined data from the sera of all E2 vaccinated animals. For both tests, a *p* value of <0.05 was considered significant. All statistical analyses were performed using Prism version 7 software.

## Data availability

Data will be shared upon request to the corresponding author, Heidi Drummer.

## Author contributions

R. J. C., P. P., and H. E. D. conceptualization; R. J. C. formal analysis; R. J. C., I. B., and H. E. D. supervision; R. J. C., I. B., L. P., and J. M. investigation; R. J. C., I. B., L. P., J. M., and H. E. D. methodology; R. J. C. and H. E. D. writing-original draft; R. J. C., I. B., J. M., P. P., and H. E. D. writing-review and editing; I. B. validation; L. P. and H. E. D. data curation; P. P. and H. E. D. funding acquisition; P. P. and H. E. D. project administration.

## Supplementary Material

Supporting Information

## Footnotes

3 H. Drummer, unpublished observation.

## Footnotes

2 The abbreviations used are:

HCV: hepatitis C virus
HMW: high-molecular-weight
NAb: neutralizing antibody
bNAb: broadly neutralizing antibody
non-NAb: nonneutralizing antibody
HVR: hypervariable region
RBD: receptor-binding domain
SEC: size-exclusion chromatography
SEC-MALS: SEC-multiangle light scattering
LEL: large extracellular loop
PBS 6.8: PBS adjusted to pH 6.8
RT: room temperature
BN-PAGE: blue native PAGE
DSF: differential scanning fluorimetry
*T_m_*: melting temperature
OD: optical density
ID_50_: inhibitory dilution 50
BLI: bilayer interferometry
HCVpp: pseudotyped retroviral particles
HCVcc: cell culture–derived virus
CMI: cell-mediated immunity
BisTris: 2-[bis(2-hydroxyethyl)amino]-2-(hydroxymethyl)propane-1,3-diol.
