# Supplementary material for: Enhancing the antigenicity and immunogenicity of monomeric forms of hepatitis C virus E2 for use as a preventive vaccine
Source: J Biol Chem. 2020 Apr 16;295(21):7179–92. doi: 10.1074/jbc.RA120.013015 (PMC7247312; doi:10.1074/jbc.RA120.013015)
Supplement: Supporting Information [file supp_295_21_7179__index.html]

Enhancing the antigenicity and immunogenicity of monomeric forms of hepatitis C virus E2 for use as a preventive vaccine — Multimerization of HCV E2 enhances immunogenicity — Enhancing the antigenicity and immunogenicity of monomeric forms of hepatitis C virus E2 for use as a preventive vaccine — EDITORS' PICK: Multimerization of HCV E2 enhances immunogenicity — Supporting Information 

# Enhancing the antigenicity and immunogenicity of monomeric forms of hepatitis C virus E2 for use as a preventive vaccine

## Supporting Information

- Supporting Information (to be published online) - Table S1 and figures S1-S4
