# Supplementary material for: Enhancing the antigenicity and immunogenicity of monomeric forms of hepatitis C virus E2 for use as a preventive vaccine
Source: J Biol Chem. 2020 Apr 16;295(21):7179–92. doi: 10.1074/jbc.RA120.013015 (PMC7247312; doi:10.1074/jbc.RA120.013015)
Supplement: Supporting Information [file supp_RA120.013015_158793_2_supp_510523_q8r4gj.pdf]

Supporting Information

Enhancing the antigenicity and immunogenicity of monomeric forms of hepatitis C virus E2 for use as a preventative vaccine

Rob J. Center, Irene Boo, Lilian Phu, Joey McGregor, Pantelis Poumbourios, Heidi E. Drummer

Material included:

Table S1
Figure S1
Figure S2
Figure S3
Figure S4

MAb	Antigen	K_D (M) x 10^{-9}	k_{on} (1/Ms) x 10^{-5}	k_{off} (1/s) x 10^{-5}	RMax
HCV1	Δ123	27.3	10.8	29.5	0.2631
	Δ123A7	2.93	83.6	24.5	0.2176
	Δ123r	ND	18.1	ND	0.2219
	Δ123A7r	ND	19.5	ND	0.1327
	Δ123-HMW	ND	12.0	ND	0.2901
AR3C	Δ123	34.9	23.5	82.1	0.4265
	Δ123A7	6.45	2.69	174	0.3590
	Δ123r	ND	14.7	ND	0.0426
	Δ123A7r	ND	845	ND	0.0999
	Δ123-HMW	ND	933	ND	0.2568
2A12	Δ123	32.2	41.5	133	0.1591
	Δ123A7	1.13	7.68	86.6	0.1227
	Δ123r	ND	11.1	ND	0.0336
	Δ123A7r	ND	11.0	ND	0.0620
	Δ123-HMW	ND	14.1	ND	0.0386

Table S1. BLI-derived binding values for the indicated MAb/antigen pairs
ND = not determined due to the absence of a detectable off rate.

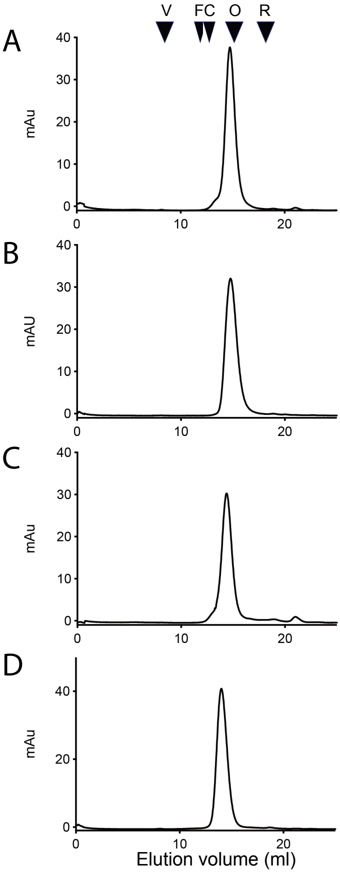

Figure S1.

Figure S1. Representative analytical SEC of pooled monomeric fractions of Δ123 (A), Δ123A7 (B), RBD (C) and RBDA7 (D). The SEC standards (abbreviation, elution volume and mass) used were Blue dextran (V = void, 8.0 ml, > 2000 kDa), Ferritin (F, 11.8 ml, 440 kDa), Catalase (C, 13.4 ml, 232 kDa) Ovalbumin (O, 15.4 ml, 43 kDa) and Ribonuclease A (R, 17.8 ml, 13.7 kDa).

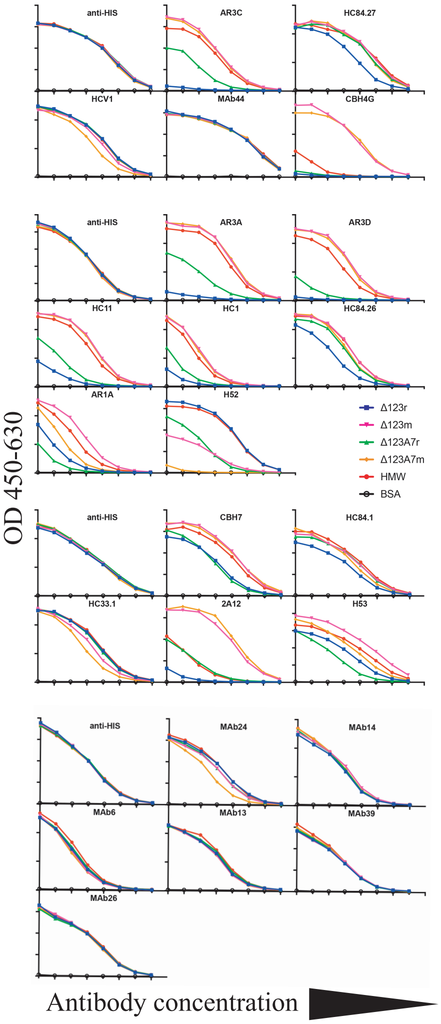
anti-HIS
AR3C
HC84.27
HCV1
MAb44
CBH4G
anti-HIS
AR3A
AR3D
HC11
HC1
HC84.26
AR1A
H52
Δ123r
Δ123m
Δ123A7r
Δ123A7m
HMW
BSA
OD 450-630
anti-HIS
CBH7
HC84.1
HC33.1
2A12
H53
anti-HIS
MAb24
MAb14
MAb6
MAb13
MAb39
MAb26
Antibody concentration

Figure S2

Figure S2.

The binding of monoclonal antibodies to plate-bound E2 antigens. Equal amounts by weight of HMW, Δ123r, Δ123A7r, Δ123m, Δ123A7m were absorbed to solid-phase plates followed by the addition of serial dilutions of each monoclonal antibody as indicated. The same amount of BSA was absorbed as a negative control. Reactivity of the E2 antigens to the anti-6 HIS tag antibody was used confirm equal absorption.

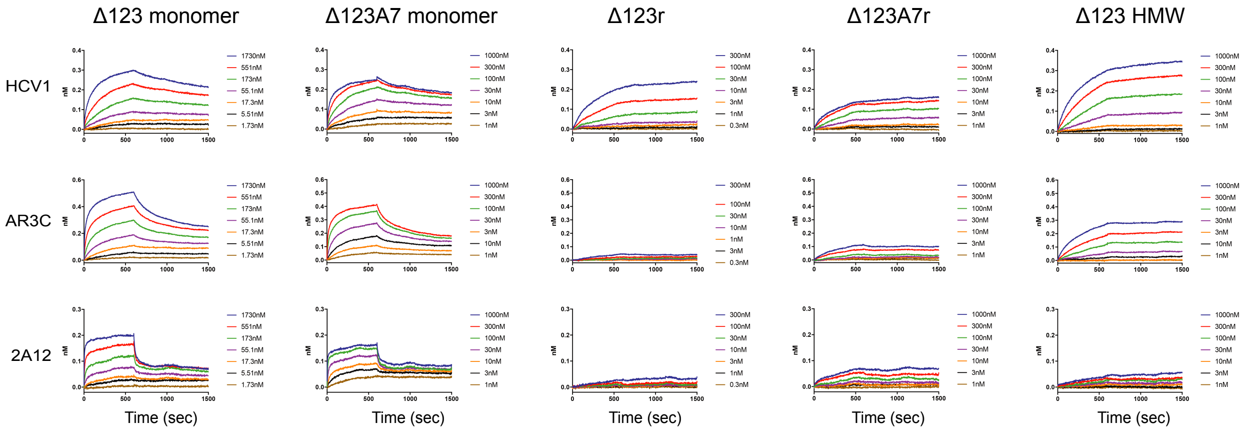

Figure S3. BLI analysis of the indicated different E2 antigens using the MAbs HCV1, AR3C and 2A12 in the top, middle and bottom rows respectively. The experimental values obtained are shown in supplementary Table 1.

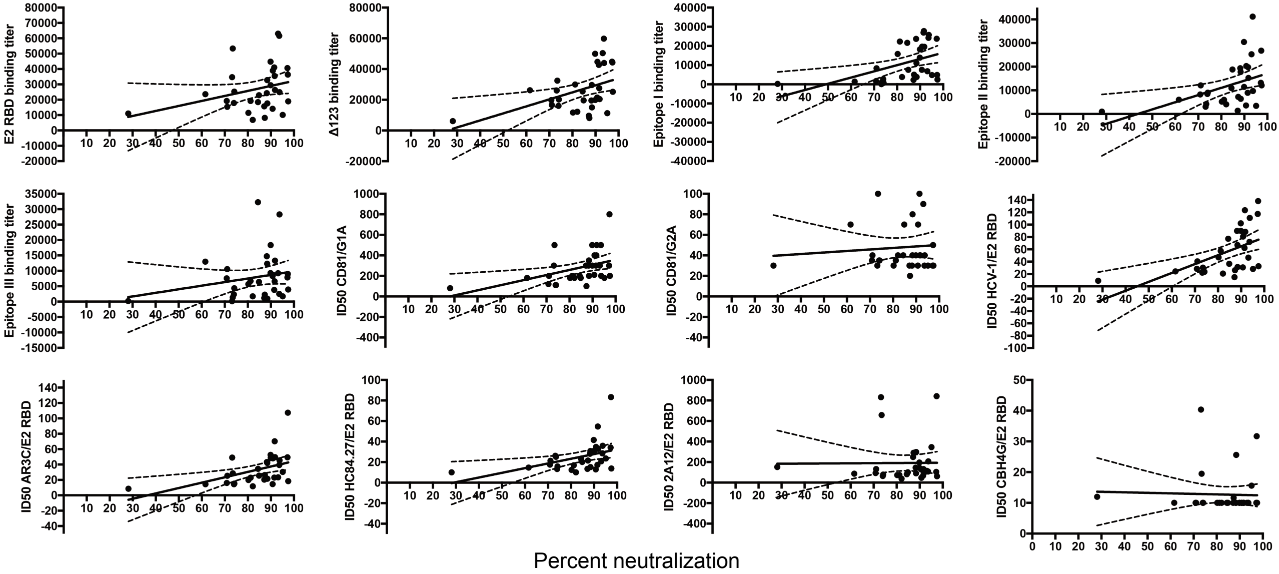

Figure S4. Correlations between the percentage of H77pp neutralization at 1:40 dilution and the indicated experimental parameters. Solid lines represent the mean linear regression and the dashed lines show the 95% confidence intervals. P and r values are shown in Table 4.
